# Crystal structure of benzo[*h*]quinoline-3-carbox­amide

**DOI:** 10.1107/S2056989019014440

**Published:** 2019-11-05

**Authors:** Christoph W. Grathwol, Nicolas Chrysochos, Benedict J. Elvers, Andreas Link, Carola Schulzke

**Affiliations:** a Institut für Pharmazie, Universität Greifswald, Friedrich-Ludwig-Jahn-Strasse 17, 17489 Greifswald, Germany; b Institut für Biochemie, Felix-Hausdorff-Strasse 4, 17489 Greifswald, Germany

**Keywords:** crystal structure, benzo­quinoline, nicotinamide derivative, photocyclization

## Abstract

The title com­pound, C_14_H_10_N_2_O, crystallizes in the monoclinic space group *P*2_1_/*c* with four mol­ecules in the unit cell. All 17 non-H atoms of one mol­ecule lie essentially in one plane. In the unit cell, two pairs of mol­ecules are exactly coplanar, while the angle between these two orientations is close to perfectly perpendicular at 87.64 (6)°. In the crystal, mol­ecules adopt a 50:50 crisscross arrangement, which is held together by two nonclassical and two classical inter­molecular hydrogen bonds. The hydrogen-bonding network together with off-centre π–π stacking inter­actions between the pyridine and outermost benzene rings, stack the mol­ecules along the *b*-axis direction.

## Abstract 2

A benzo­quinone com­pound was unintentionally synthesized by photocyclization and subsequent oxidation in air while attempting to transform the *E* isomer of a nicotinamide derivative to its *Z* isomer. The chemical and mol­ecular structures of the product was established crystallographically. The surprising synthesis, chromatographic purification and com­prehensive characterization of this com­pound including single crystal structural analysis are reported and its structure including crystal packing is discussed in detail.

## Chemical context   

Quinoline and benzo­quinoline scaffolds are common structural motifs in artificial, as well as natural products, and many of these com­pounds are of enormous value for pharmacotherapy. Their multifaceted biological efficacy is outstanding and ranges from cardiovascular (Ferlin *et al.*, 2002 ▸; Abouzid *et al.*, 2008 ▸) and anti-inflammatory effects (Kumar *et al.*, 2009 ▸; Hussaini, 2016 ▸) to anti­microbial (El Shehry *et al.*, 2018 ▸), as well as anti­cancer activity (Abdelsalam *et al.*, 2019 ▸; Haiba *et al.*, 2019 ▸; Jafari *et al.*, 2019 ▸; Musiol, 2017 ▸; Marzaro *et al.*, 2016 ▸). In a report on 3-(tetra­zol-5-yl)quinolines with anti­allergic potential, benzo[*h*]quinoline-3-carboxamide was mentioned as a synthetic inter­mediate, though its biological activity was not determined in that work (Erickson *et al.*, 1979 ▸). In our recent studies on photoswitchable sirtuin inhibitors, we obtained benzo[*h*]quinoline-3-carboxamide as a side product of aza­stilbene photoisomerization (Grathwol *et al.*, 2019 ▸). By UV radiation, (*E*)-5-styrylnicotinamide was transformed to its *Z* isomer as envisioned, but underwent photocyclization and successive oxidation, yielding two isomeric benzo­quinoline derivatives; the identity of one of these was determined to be the benzo[*h*]quinoline derivative and its crystal structure is reported here.
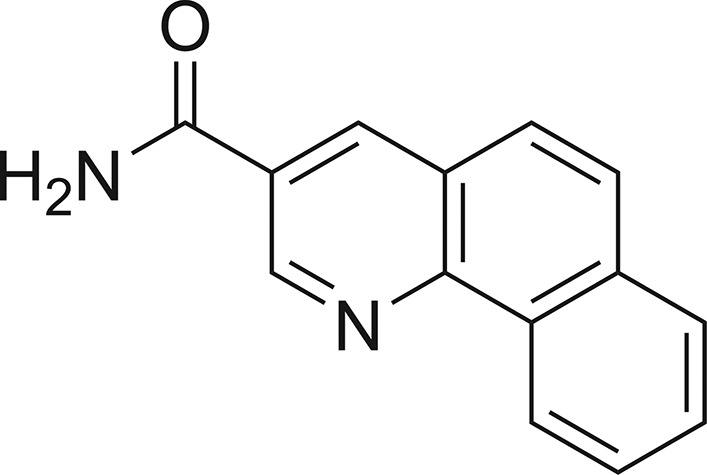


## Structural commentary   

**Figure 1:**
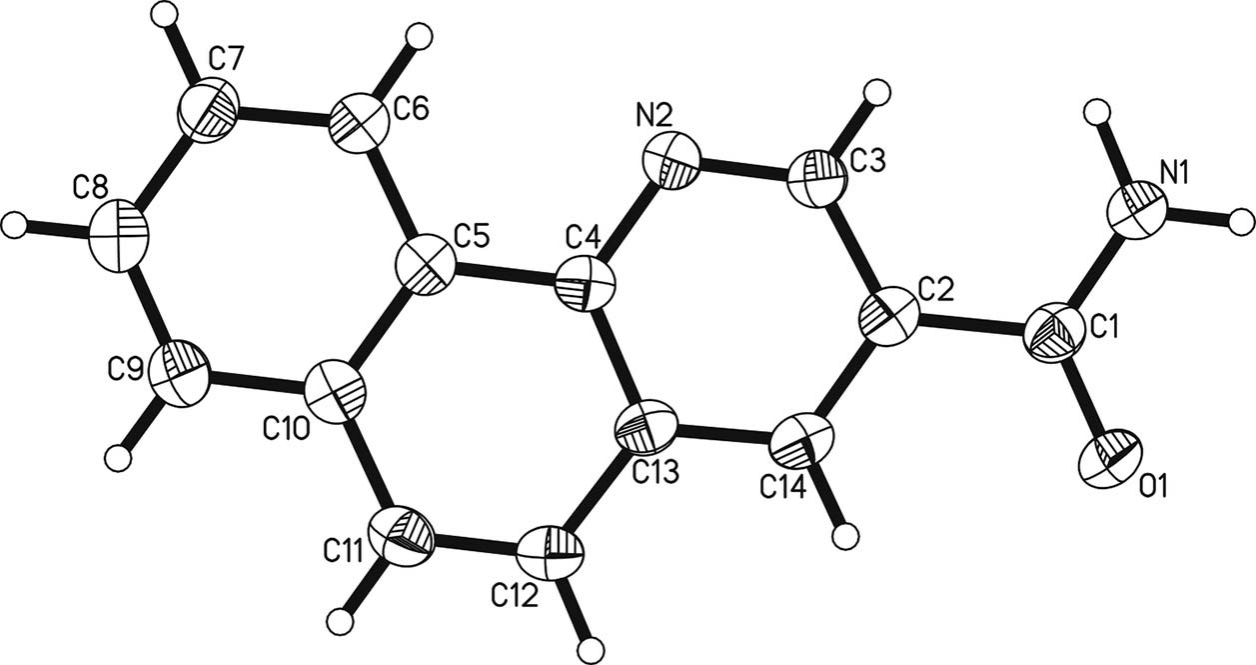
The mol­ecular structure of benzo[*h*]quinoline-3-carboxamide. Displacement ellipsoids are shown at the 50% probability level.

**Figure 2:**
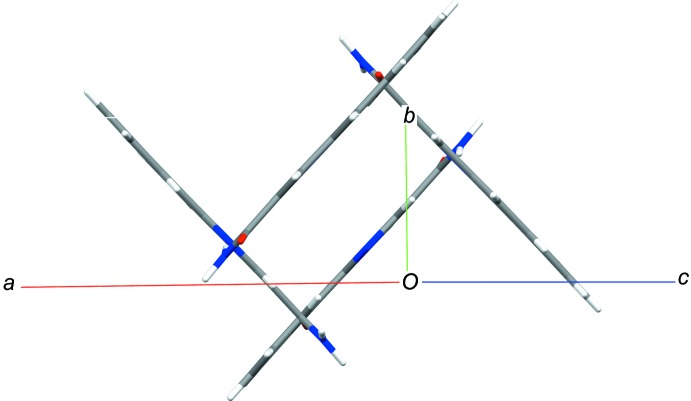
The unit cell of benzo[*h*]quinoline-3-carboxamide in *P*2_1_/*c*, with its four mol­ecules in a coplanar and perpendicular arrangement, viewed along the *ac* diagonal.

The title com­pound, benzo[*h*]quinoline-3-carboxamide, crystallizes in the monoclinic space group *P*2_1_/*c*. Four mol­ecules are present in the unit cell (*Z* = 4) and there is one mol­ecule in the asymmetric unit. Benzo[*h*]quinoline-3-carboxamide consists of a nicotinamide unit being fused with a benzo[*h*]quinoline moiety, while the pyridine ring is shared between these two common structural building blocks (Fig. 1 ▸). The mol­ecule is essentially flat, with a largest deviation from the plane through all 17 non-H atoms of 0.050 (2) Å (O1) and an r.m.s. deviation of 0.020 (2) Å. In the unit cell, the four mol­ecules are arranged in two perfectly coplanar pairs, with a nearly perpendicular angle between the respective planes of the two pairs of 87.64 (6)° (Fig. 2 ▸). A plethora of crystal structures are known for com­pounds with one or other of the two building blocks that make up this mol­ecule [for the nicotinamide scaffold, *ConQuest* finds over 2000 hits in the Cambridge Structural Database (CSD), while for benzo­quinoline, there are over 500; Groom *et al.*, 2016 ▸]. However, the specific combination in the title com­pound is unprecedented. Comparing the title com­pound to the known structures of unsubstituted nicotinamides, its pronounced planarity is most notable. In the six published structures in the space groups *P*2_1_/*c* or *P*2_1_/*a*, the angles between the aromatic plane (here C2/C3/N2/C4/C13/C14) and the amide substituent (here O1/N1/C1) range from 22.1 to 23.3° (general CSD refcode NICOAM; Wright & King, 1954 ▸; Miwa *et al.*, 1999 ▸; Fábián *et al.*, 2011 ▸; Jarzembska *et al.*, 2014 ▸), *i.e.* this angle is quite consistent. In the only distinct polymorph of a nicotinamide in the space group *P*2/*a*, four distinct mol­ecules were refined with this angle ranging from 8.1 to 22.4° (Li *et al.*, 2011 ▸), *i.e.* they are not very consistent but still considerably larger than the corresponding angle found in the title com­pound, which is a mere 3.3 (4)°. This points toward an extension of the aromatic resonance systems to include the amide substituent. In the parent nicotinamide scaffolds, this does not occur. Similarly, the com­paratively long C1=O1 distance of 1.238 (3) Å (average 1.23 Å) and the com­paratively short C1—C2 distance of 1.491 (3) Å (average 1.50 Å in other nicotinamide structures) indicate some involvement of these atoms in resonance effects. In support of this extended resonance, in the nicotinamide structures, the aromatic C—C bonds are much less diverse (range 1.38–1.39 Å, indicating very strong aromaticity in the pyridine ring) than in the structure reported here. In fact, the C—C [range 1.376 (3)–1.414 (3) Å] and C—N [1.321 (3) and 1.360 (3) Å] bond lengths here are much more similar to the two known structures of 2-unsubstituted and 3-substituted benzo[*h*]quinolines (refcodes JAFVEU and SUDVES), with ranges of average C—C and C—N bond lengths of 1.38–1.42 and 1.32–1.36 Å, respectively (Martínez *et al.*, 1992 ▸; Luo *et al.*, 2015 ▸). The benzo[*h*]quinoline structural motif therefore dominates the observed metrical parameters of the mol­ecule reported here, representing a fusion between a nicotinamide and a benzo[*h*]quinoline, with a partial extension of the aromaticity beyond the ring system and extending towards the amide substituent.

## Supra­molecular features   

**Figure 3:**
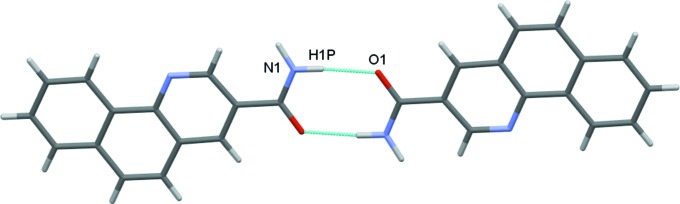
Dimers formed by N—H⋯O hydrogen bonds.

**Figure 4:**
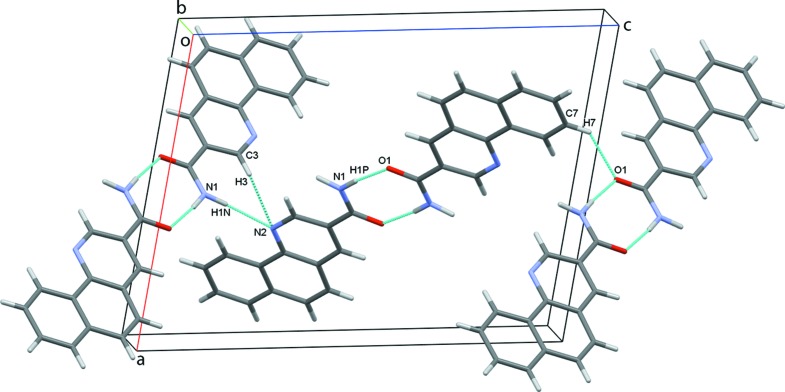
Chains of dimers along the *c*-axis.

**Figure 5:**
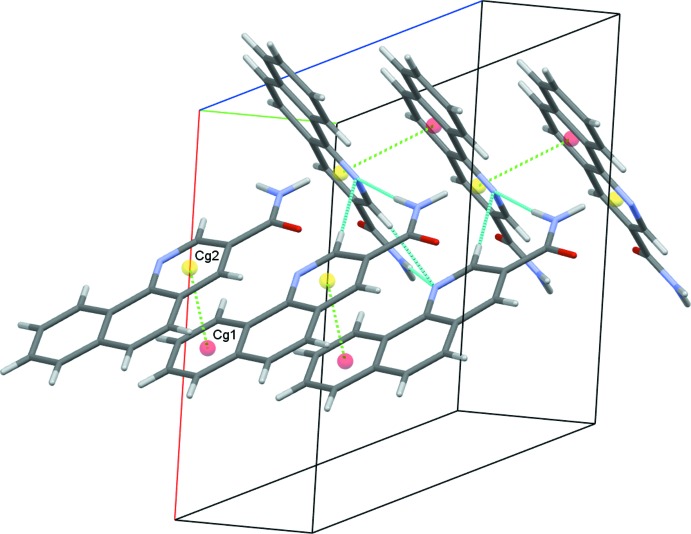
π–π stacking inter­actions, with centroids shown as coloured spheres. *Cg*1 and *Cg*2 are the centroids of the C5–C10 and C2/C3/N2/C4/C13/C14 rings, respectively.

**Figure 6:**
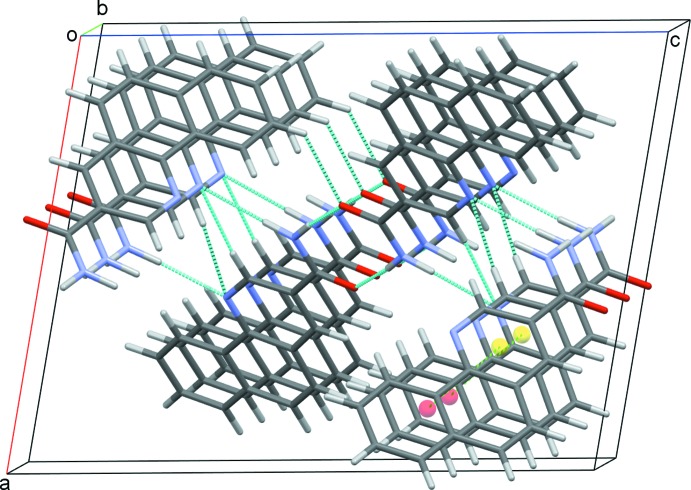
The overall packing of the title com­pound, viewed along the *b*-axis direction.

In the crystal, the planar mol­ecules are all arranged in planes in two distinct orientations, which are nearly perpendicular to each other [angle 87.64 (6)°]. This forms a crisscross pattern when viewed along the *ac* diagonal (Fig. 2 ▸). Classical inversion-related N1—H1*P*⋯O1 hydrogen bonds form dimers and generate 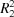
(8) ring motifs (Fig. 3 ▸). Each mol­ecule forms two classical (N—H⋯O and N—H⋯N) and two nonclassical (C—H⋯N and C—H⋯O) hydrogen bonds (Table 1 ▸), and these contacts link adjacent dimers into zigzag chains along the *c*-axis direction (Fig. 4 ▸). The observed packing is further stabilized by off-centre π–π stacking between the pyridine and outermost benzene rings of each of the coplanar layers [centroid-to-centroid distance = 3.610 (1) Å] (Fig. 5 ▸). These contacts combine to stack the mol­ecules along the *b*-axis direction (Fig. 6 ▸).

## Synthesis and crystallization   

A solution of (*E*)-5-styrylnicotinamide (673 mg, 3.00 mmol, 1.00 equiv.) in methanol (350 ml) was treated with a solution of iodine (38 mg, 0.15 mmol, 0.05 equiv.) in methanol (50 ml). A slow stream of com­pressed air was bubbled through the reaction mixture while it was irradiated with UV light (six Vilber-Lourmat T8-C lamps, 8 W, 254 nm). After com­plete consumption of the starting material (24 h), the solvent was removed under reduced pressure. Purification of the residue by silica-gel column chromatography (*n*-hexa­ne/THF, 1:1 *v*/*v*) gave pure benzo[*h*]quinoline-3-carboxamide as a colourless solid (yield 80 mg, 0.36 mmol, 12%). Crystallization was accom­plished by slow evaporation of a solution in THF (5 mg ml^−1^) and yielded the title com­pound as colourless needles: *R*_F_ = 0.32 (*n*-hexa­ne/THF, 1:1 *v*/*v*); m.p. 549.8 K (decom­position); ^1^H NMR, H,H-COSY (400 MHz, DMSO-*d*_6_): δ (ppm) 9.48 (*d*, *J* = 2.2 Hz, 1H, C3-H), 9.26–9.19 (*m*, 1H, C6-H), 8.89 (*d*, *J* = 2.1 Hz, 1H, C14-H), 8.36 (*s*, *br*, 1H, N1-H), 8.12–8.07 (*m*, 1H, C9-H), 8.03 (*d*, *J* = 8.9 Hz, 1H, C11-H), 7.95 (*d*, *J* = 8.9 Hz, 1H, C12-H), 7.85–7.78 (*m*, 2H, C7-H, C8-H), 7.74 (*s*, *br*, 1H, N1-H); ^13^C NMR, DEPT135, HSQC, HMBC (101 MHz, DMSO-*d*_6_): δ (ppm) 166.4 (C1), 147.8 (C3), 146.7 (C4), 135.5 (C14), 133.8 (C13), 130.3 (C5), 129.0 (C8), 128.1 (C9/C11), 128.0 (C9/C11), 127.8 (C2), 127.3 (C7), 125.8 (C12), 124.9 (C10), 124.1 (C6); IR (ATR): ν (cm^−1^) 3336, 3136, 1686, 1482, 1395, 1295, 801, 691, 539, 489; ESI–HRMS calculated for [C_14_H_10_N_2_O + H]^+^ 222.0793, found 222.0796; compound purity (220 nm): 100%.

## Refinement   

Crystal data, data collection and structure refinement details are summarized in Table 2 ▸. All C-bound H atoms constitute aromatic protons, which were attached in calculated positions and treated as riding with *U*_iso_(H) = 1.2*U*_eq_(C). The two amine H atoms were found and refined without any constraints or restraints.

## Supplementary Material

Crystal structure: contains datablock(s) I. DOI: 10.1107/S2056989019014440/sj5580sup1.cif

Structure factors: contains datablock(s) I. DOI: 10.1107/S2056989019014440/sj5580Isup2.hkl

Supporting information file. DOI: 10.1107/S2056989019014440/sj5580Isup3.cml

CCDC references: 1960760, 1960760

Additional supporting information:  crystallographic information; 3D view; checkCIF report

## Figures and Tables

**Table 1:** Hydrogen-bond geometry (Å, °)

*D*—H⋯*A*	*D*—H	H⋯*A*	*D*⋯*A*	*D*—H⋯*A*
N1—H1*N*⋯N2^i^	0.97 (3)	2.17 (3)	3.133 (3)	173 (2)
N1—H1*P*⋯O1^ii^	0.93 (3)	1.96 (3)	2.895 (3)	175 (3)
C3—H3⋯N2^i^	0.95	2.41	3.361 (3)	174
C7—H7⋯O1^iii^	0.95	2.45	3.140 (3)	129

Symmetry codes: (i) 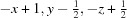; (ii) 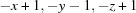; (iii) 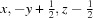.

**Table 2:** Experimental details

Crystal data	
Chemical formula	C_14_H_10_N_2_O
*M*_r_	222.24
Crystal system, space group	Monoclinic, *P*2_1_/*c*
Temperature (K)	170
*a*, *b*, *c* (Å)	12.634 (3), 4.9426 (10), 16.778 (3)
β (°)	100.53 (3)
*V* (Å^3^)	1030.0 (4)
*Z*	4
Radiation type	Mo *K*α
μ (mm^−1^)	0.09
Crystal size (mm)	0.37 × 0.07 × 0.04

Data collection	
Diffractometer	Stoe IPDS-2T
Absorption correction	Numerical face indexed
*T*_min_, *T*_max_	0.727, 0.997
No. of measured, independent and observed [*I* > 2σ(*I*)] reflections	10053, 2551, 1320
*R*_int_	0.087
(sin θ/λ)_max_ (Å^−1^)	0.667

Refinement	
*R*[*F* ^2^ > 2σ(*F* ^2^)], *wR*(*F* ^2^), *S*	0.055, 0.167, 0.98
No. of reflections	2551
No. of parameters	163
H-atom treatment	H atoms treated by a mixture of independent and constrained refinement
Δρ_max_, Δρ_min_ (e Å^−3^)	0.23, −0.27

Computer programs: *X-AREA* (Stoe & Cie, 2010 ▸), *SHELXT2018* (Sheldrick, 2015*a* ▸), *SHELXL2018* (Sheldrick, 2015*b* ▸), *XP* in *SHELXTL* (Sheldrick, 2008 ▸), *Mercury* (Macrae *et al.*, 2008 ▸), *CIFTAB* (Sheldrick, 2015*b* ▸) and *publCIF* (Westrip, 2010 ▸).

## supplementary crystallographic information

### Crystal data

**Table:**

C_14_H_10_N_2_O	*F*(000) = 464
*M**_r_* = 222.24	*D*_x_ = 1.433 Mg m^−^^3^
Monoclinic, *P*2_1_/*c*	Mo *K*α radiation, λ = 0.71073 Å
*a* = 12.634 (3) Å	Cell parameters from 11960 reflections
*b* = 4.9426 (10) Å	θ = 6.4–59.0°
*c* = 16.778 (3) Å	µ = 0.09 mm^−^^1^
β = 100.53 (3)°	*T* = 170 K
*V* = 1030.0 (4) Å^3^	Needle, colourless
*Z* = 4	0.37 × 0.07 × 0.04 mm

### Data collection

**Table:**

Stoe IPDS2T diffractometer	2551 independent reflections
Radiation source: fine-focus sealed tube	1320 reflections with *I* > 2σ(*I*)
Detector resolution: 6.67 pixels mm^-1^	*R*_int_ = 0.087
ω scans	θ_max_ = 28.3°, θ_min_ = 3.2°
Absorption correction: numerical face indexed	*h* = −16→16
*T*_min_ = 0.727, *T*_max_ = 0.997	*k* = −6→6
10053 measured reflections	*l* = −22→22

### Refinement

**Table:**

Refinement on *F*^2^	Secondary atom site location: difference Fourier map
Least-squares matrix: full	Hydrogen site location: mixed
*R*[*F*^2^ > 2σ(*F*^2^)] = 0.055	H atoms treated by a mixture of independent and constrained refinement
*wR*(*F*^2^) = 0.167	*w* = 1/[σ^2^(*F*_o_^2^) + (0.086*P*)^2^] where *P* = (*F*_o_^2^ + 2*F*_c_^2^)/3
*S* = 0.98	(Δ/σ)_max_ < 0.001
2551 reflections	Δρ_max_ = 0.23 e Å^−^^3^
163 parameters	Δρ_min_ = −0.27 e Å^−^^3^
0 restraints	Extinction correction: SHELXL2018 (Sheldrick, 2015b), Fc^*^=kFc[1+0.001xFc^2^λ^3^/sin(2θ)]^-1/4^
Primary atom site location: dual	Extinction coefficient: 0.019 (4)

### Special details

**Table:**

Geometry. All esds (except the esd in the dihedral angle between two l.s. planes) are estimated using the full covariance matrix. The cell esds are taken into account individually in the estimation of esds in distances, angles and torsion angles; correlations between esds in cell parameters are only used when they are defined by crystal symmetry. An approximate (isotropic) treatment of cell esds is used for estimating esds involving l.s. planes.

### Fractional atomic coordinates and isotropic or equivalent isotropic displacement parameters (Å^2^)

**Table:**

	*x*	*y*	*z*	*U*_iso_*/*U*_eq_	
O1	0.60103 (14)	−0.2438 (3)	0.51392 (9)	0.0364 (4)	
N2	0.63400 (15)	0.2758 (4)	0.28016 (11)	0.0304 (5)	
N1	0.48278 (17)	−0.3250 (4)	0.39872 (12)	0.0331 (5)	
C1	0.56558 (19)	−0.1907 (4)	0.44191 (13)	0.0312 (5)	
C2	0.61839 (18)	0.0252 (4)	0.40098 (12)	0.0287 (5)	
C3	0.58725 (19)	0.0909 (4)	0.31880 (13)	0.0311 (5)	
H3	0.528010	−0.004871	0.288573	0.037*	
C4	0.71867 (17)	0.4149 (4)	0.32278 (12)	0.0274 (5)	
C5	0.77008 (18)	0.6182 (4)	0.28123 (13)	0.0292 (5)	
C6	0.7362 (2)	0.6735 (5)	0.19826 (13)	0.0332 (5)	
H6	0.678537	0.573337	0.167687	0.040*	
C7	0.7858 (2)	0.8702 (5)	0.16151 (14)	0.0360 (6)	
H7	0.762331	0.905703	0.105414	0.043*	
C8	0.8707 (2)	1.0200 (5)	0.20521 (15)	0.0375 (6)	
H8	0.904089	1.157365	0.178843	0.045*	
C9	0.90571 (19)	0.9702 (5)	0.28530 (14)	0.0346 (6)	
H9	0.963477	1.073076	0.314661	0.041*	
C10	0.85689 (18)	0.7665 (4)	0.32529 (13)	0.0305 (5)	
C11	0.8935 (2)	0.7074 (5)	0.40961 (14)	0.0350 (6)	
H11	0.952662	0.805664	0.438992	0.042*	
C12	0.84594 (19)	0.5161 (5)	0.44794 (13)	0.0329 (5)	
H12	0.872211	0.481242	0.503736	0.039*	
C13	0.75657 (17)	0.3646 (4)	0.40605 (12)	0.0291 (5)	
C14	0.70365 (19)	0.1668 (4)	0.44428 (13)	0.0307 (5)	
H14	0.726853	0.130640	0.500358	0.037*	
H1N	0.449 (2)	−0.279 (6)	0.344 (2)	0.059 (9)*	
H1P	0.452 (2)	−0.464 (6)	0.4246 (16)	0.054 (8)*	

### Atomic displacement parameters (Å^2^)

**Table:**

	*U*^11^	*U*^22^	*U*^33^	*U*^12^	*U*^13^	*U*^23^
O1	0.0445 (10)	0.0389 (9)	0.0247 (8)	−0.0018 (8)	0.0036 (7)	0.0056 (7)
N2	0.0311 (11)	0.0302 (10)	0.0293 (9)	−0.0018 (8)	0.0037 (8)	0.0005 (8)
N1	0.0373 (12)	0.0331 (11)	0.0280 (10)	−0.0018 (9)	0.0036 (9)	0.0020 (8)
C1	0.0351 (13)	0.0302 (11)	0.0285 (11)	0.0031 (10)	0.0064 (10)	0.0001 (9)
C2	0.0310 (12)	0.0281 (11)	0.0275 (10)	0.0048 (9)	0.0068 (9)	0.0004 (9)
C3	0.0333 (12)	0.0306 (11)	0.0286 (11)	−0.0012 (10)	0.0033 (9)	0.0002 (9)
C4	0.0278 (11)	0.0261 (11)	0.0279 (11)	0.0029 (9)	0.0036 (9)	−0.0021 (9)
C5	0.0299 (12)	0.0276 (11)	0.0307 (11)	0.0034 (10)	0.0068 (9)	−0.0007 (9)
C6	0.0345 (13)	0.0355 (13)	0.0295 (11)	−0.0024 (10)	0.0058 (10)	0.0009 (9)
C7	0.0372 (14)	0.0387 (13)	0.0327 (11)	−0.0005 (11)	0.0082 (10)	0.0042 (10)
C8	0.0367 (13)	0.0348 (13)	0.0428 (13)	−0.0015 (11)	0.0124 (11)	0.0025 (11)
C9	0.0325 (13)	0.0324 (12)	0.0393 (13)	0.0003 (10)	0.0079 (10)	−0.0006 (10)
C10	0.0291 (12)	0.0280 (11)	0.0351 (11)	0.0030 (9)	0.0079 (9)	−0.0021 (9)
C11	0.0319 (13)	0.0379 (13)	0.0342 (12)	−0.0024 (10)	0.0037 (10)	−0.0063 (10)
C12	0.0324 (12)	0.0372 (12)	0.0274 (11)	0.0009 (10)	0.0009 (9)	−0.0027 (9)
C13	0.0303 (12)	0.0302 (11)	0.0260 (10)	0.0045 (10)	0.0033 (9)	0.0006 (9)
C14	0.0352 (13)	0.0312 (12)	0.0248 (10)	0.0053 (10)	0.0034 (9)	0.0009 (9)

### Geometric parameters (Å, º)

**Table:**

O1—C1	1.238 (3)	C6—H6	0.9500
N2—C3	1.321 (3)	C7—C8	1.396 (3)
N2—C4	1.360 (3)	C7—H7	0.9500
N1—C1	1.335 (3)	C8—C9	1.358 (3)
N1—H1N	0.97 (3)	C8—H8	0.9500
N1—H1P	0.93 (3)	C9—C10	1.413 (3)
C1—C2	1.491 (3)	C9—H9	0.9500
C2—C14	1.376 (3)	C10—C11	1.436 (3)
C2—C3	1.401 (3)	C11—C12	1.346 (3)
C3—H3	0.9500	C11—H11	0.9500
C4—C13	1.414 (3)	C12—C13	1.428 (3)
C4—C5	1.443 (3)	C12—H12	0.9500
C5—C6	1.406 (3)	C13—C14	1.404 (3)
C5—C10	1.411 (3)	C14—H14	0.9500
C6—C7	1.364 (3)		
			
C3—N2—C4	118.11 (19)	C6—C7—H7	119.6
C1—N1—H1N	124.6 (17)	C8—C7—H7	119.6
C1—N1—H1P	117.5 (17)	C9—C8—C7	120.2 (2)
H1N—N1—H1P	118 (2)	C9—C8—H8	119.9
O1—C1—N1	122.2 (2)	C7—C8—H8	119.9
O1—C1—C2	119.3 (2)	C8—C9—C10	120.5 (2)
N1—C1—C2	118.55 (19)	C8—C9—H9	119.7
C14—C2—C3	117.0 (2)	C10—C9—H9	119.7
C14—C2—C1	119.62 (19)	C5—C10—C9	119.1 (2)
C3—C2—C1	123.4 (2)	C5—C10—C11	119.4 (2)
N2—C3—C2	125.0 (2)	C9—C10—C11	121.5 (2)
N2—C3—H3	117.5	C12—C11—C10	121.5 (2)
C2—C3—H3	117.5	C12—C11—H11	119.3
N2—C4—C13	121.5 (2)	C10—C11—H11	119.3
N2—C4—C5	118.57 (19)	C11—C12—C13	121.0 (2)
C13—C4—C5	119.92 (19)	C11—C12—H12	119.5
C6—C5—C10	119.0 (2)	C13—C12—H12	119.5
C6—C5—C4	122.1 (2)	C14—C13—C4	118.1 (2)
C10—C5—C4	118.95 (19)	C14—C13—C12	122.68 (19)
C7—C6—C5	120.3 (2)	C4—C13—C12	119.3 (2)
C7—C6—H6	119.9	C2—C14—C13	120.37 (19)
C5—C6—H6	119.9	C2—C14—H14	119.8
C6—C7—C8	120.9 (2)	C13—C14—H14	119.8

### Hydrogen-bond geometry (Å, º)

**Table:**

*D*—H···*A*	*D*—H	H···*A*	*D*···*A*	*D*—H···*A*
N1—H1*N*···N2^i^	0.97 (3)	2.17 (3)	3.133 (3)	173 (2)
N1—H1*P*···O1^ii^	0.93 (3)	1.96 (3)	2.895 (3)	175 (3)
C3—H3···N2^i^	0.95	2.41	3.361 (3)	174
C7—H7···O1^iii^	0.95	2.45	3.140 (3)	129

Symmetry codes: (i) −*x*+1, *y*−1/2, −*z*+1/2; (ii) −*x*+1, −*y*−1, −*z*+1; (iii) *x*, −*y*+1/2, *z*−1/2.
